# The complete chloroplast genome sequence of *Ulmus szechuanica* (Ulmaceae) and its phylogenetic analysis

**DOI:** 10.1080/23802359.2020.1768958

**Published:** 2020-05-27

**Authors:** Yan Shufang, Liu Yichao, Feng Shuxiang, Huang Xiaoxu, Huang Yinran

**Affiliations:** a Hebei Academic of Forestry and Grassland, Shijiazhuang, China; b Hebei Engineering Research Center for Trees Varieties, Shijiazhuang, China; c Hebei Agricultural University, Baoding, China

**Keywords:** Chloroplast genome, *U. szechuanica*, phylogenetic

## Abstract

*Ulmus szechuanica* is a species of Sect.Ulmus and Ser.Nitentes in Ulmaceae, and it is an endangered wild plant in China. The complete chloroplast genome (cp) of *U. szechuanica* was reported in this study. The result showed that the cp genome was 159,703 bp in length including a large single-copy (LSC) 88,039 bp and a small single-copy (SSC) 19,072 bp, which were separated by two inverted repeats (IRs) of 26,296 bp with the typical quadripartite structure, respectively. The genome encoded 131 genes, including 86 protein-coding genes, 37 tRNA genes, and 8 rRNA genes. The GC content was 35.53%. Chloroplast sequences were used for constructing phylogenetic tree to determine the evolutionary status of *U. szechuanica*. The maximum-likelihood phylogenetic analysis showed that *U. szechuanica* displayed a closer kinship to five other Ulmus species. This study provides important information for identification and conservation of species, germplasm resources utilization, and genetic engineering of Ulmus. The cp will provide a reference for future studies on species evolution of Ulmus.

Being organelles for photosynthesis present in higher plants and algae, chloroplasts have played important roles in plant evolution. Chloroplast genome is very conservative, mainly in the genome structure, gene sequence and gene types (Jansen and Ruhlman 2012; Du et al. 2018). The valuable chloroplast genomic information has been utilized in investigating plant evolution, diversity and phylogenetic relationships between different species (Mu et al. 2020; Liu et al. 2020).*U. szechuanica* belongs to Ulmus under Ulmaceae. It is a deciduous tree with rapid growth and excellent material. It is one of the trees used for timber and landscape greening in China. Due to its limited provenance, poor ability of natural renewal, environmental degradation and man-made damage, the natural distribution of this species is limited. It is an endangered wild plant in China.

Fresh and clean leaves of were collected from Hang Zhou Botanical Garden in China. The voucher specimen was deposited in the herbarium of Hebei Academic of Forestry and Grassland File number：HAFG22U370). The experimental plants were planted in the Hebei Academic of Forestry and Grassland, Shijiazhuang, China (114°28′12″E, 38°08′23″N). The total genomic DNA was extracted and used for sequencing, assembly and annotation at Beijing Zhongxing Bomai Technology Co., LTD. Plant DNA extraction kit (TIANGEN, Beijing) was used to extract the total DNA of fresh young leaves. The purity, integrity and concentration of DNA were detected by agarose gel electrophoresis and NanoDrop2000 microspectrophotometry, respectively. Illumina NovaSep platform was used for sequencing after meeting the sequencing requirements. The raw data were used to denovo assemble the complete cp genome using SOAPdenovo software. The original data was filtered to obtain high-quality data. OGDRAW software (Lohse et al. 2013) was used to draw the physical map of chloroplast genome (uploaded to NCBI with the number of MT424764).

The cp genome of *U. szechuanica* is 159,703 bp in size with the typical quadripartite structure of angiosperms, consisting of two single-copy regions (LSC of 88,039 bp and SSC of 19,072 bp) and a pair of IRs regions of 26,296 bp. The total guanine-cytosine content was 35.53%. The genome contained 131 genes, including 86 protein-coding genes, 37 tRNA genes, and eight rRNA genes. Six protein-coding genes, seven tRNA genes and four rRNA genes were duplicated.

The completed cp genome sequences of *U. szechuanica* and another fourteen plant species (including five *Ulmus* species, one *Pteroceltis* specie,three *Acer* species, one *Morus* specie, one *Ficus* specie, and two *Broussonetia* species, and a outgroup *Arabidopsis thaliana*,) were used to analyze the phylogenetic position of *U. szechuanica*. The phylogenetic tree was constructed. The result showed that *U. szechuanica* was clustered with five other *Ulmus* as shown in Figure 1.

**Figure 1.**
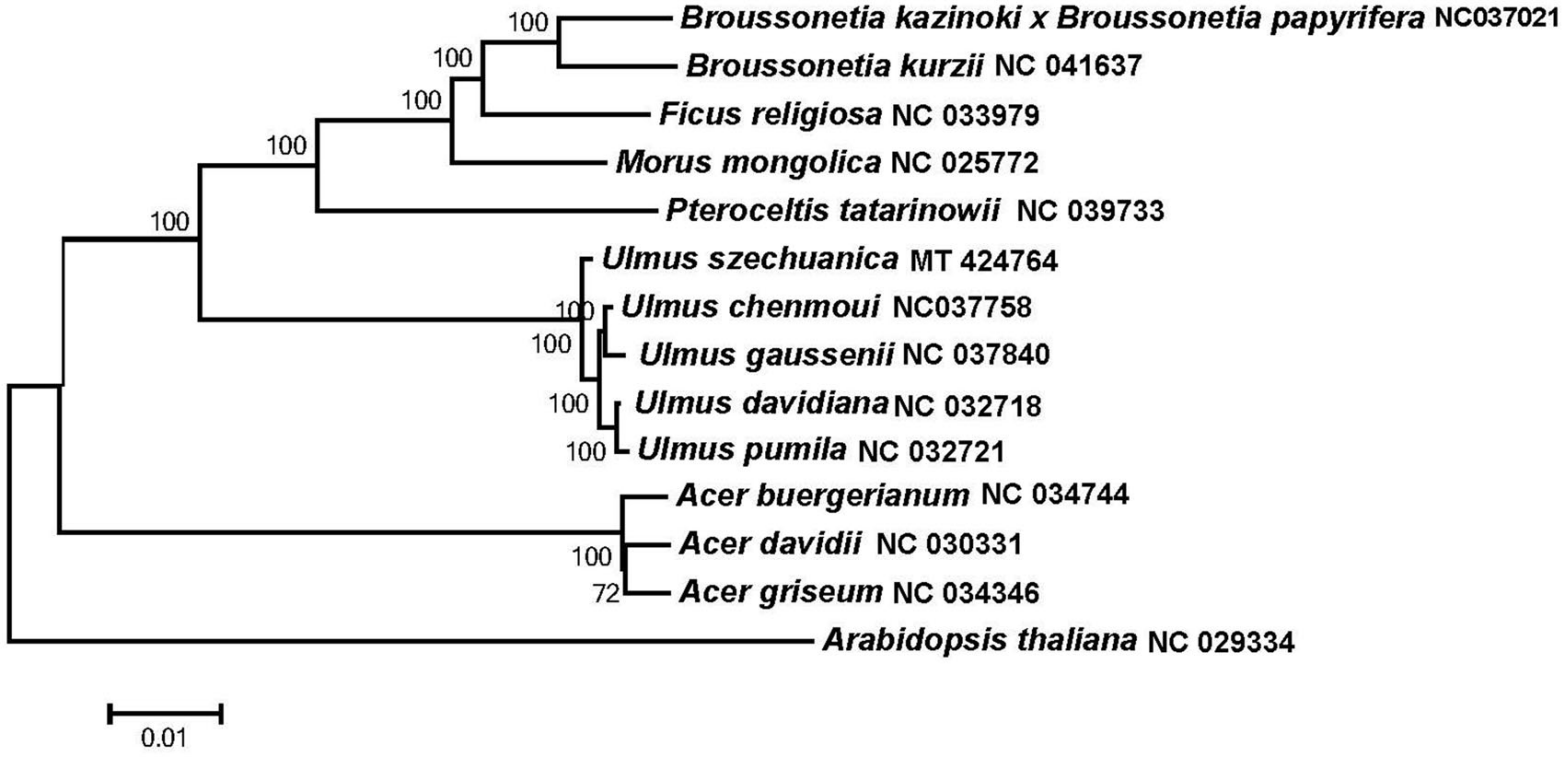
Maximum likelihood phylogenetic tree based on 14 selected plants chloroplast genome sequences.

## Data Availability

The data that support the findings of this study are openly available in figshare at http://doi.org/10.6084/m9.figshare, reference number https://doi.org/10.1016/j.ympev.2020.106784.
